# The Influence of Low Doses of Zearalenone and T-2 Toxin on Calcitonin Gene Related Peptide-Like Immunoreactive (CGRP-LI) Neurons in the ENS of the Porcine Descending Colon

**DOI:** 10.3390/toxins9030098

**Published:** 2017-03-10

**Authors:** Krystyna Makowska, Kazimierz Obremski, Lukasz Zielonka, Slawomir Gonkowski

**Affiliations:** 1 Department of Clinical Physiology, Faculty of Veterinary Medicine, University of Warmia and Mazury in Olsztyn, ul. Oczapowskiego 13, 10-718 Olsztyn, Poland; krystyna.makowska@uwm.edu.pl; 2 Department of Veterinary Prevention and Feed Hygiene, Faculty of Veterinary Medicine, University of Warmia and Mazury in Olsztyn, ul. Oczapowskiego 13, 10-718 Olsztyn, Poland; kazimierz.obremski@uwm.edu.pl (K.O.); lukasz.zielonka@uwm.edu.pl (L.Z.)

**Keywords:** CGRP, zearalenone, T-2 toxin, ENS, descending colon, pig

## Abstract

The enteric nervous system (ENS) can undergo adaptive and reparative changes in response to physiological and pathological stimuli. These manifest primarily as alterations in the levels of active substances expressed by the enteric neuron. While it is known that mycotoxins can affect the function of the central and peripheral nervous systems, knowledge about their influence on the ENS is limited. Therefore, the aim of the present study was to investigate the influence of low doses of zearalenone (ZEN) and T-2 toxin on calcitonin gene related peptide-like immunoreactive (CGRP-LI) neurons in the ENS of the porcine descending colon using a double immunofluorescence technique. Both mycotoxins led to an increase in the percentage of CGRP-LI neurons in all types of enteric plexuses and changed the degree of co-localization of CGRP with other neuronal active substances, such as substance P, galanin, nitric oxide synthase, and cocaine- and amphetamine-regulated transcript peptide. The obtained results demonstrate that even low doses of ZEN and T-2 can affect living organisms and cause changes in the neurochemical profile of enteric neurons.

## 1. Introduction

Mycotoxins are secondary metabolites produced by some fungal species. These substances exert a deleterious impact on living organisms [1]. Their effects are carcinogenic, mutagenic, estrogenic and/or neurotoxic in nature, and can lead to an acute and chronic poisoning, allergies, as well as cause a damage to internal organs (primarily liver and kidneys). Given their effects and a high resistance to temperature [1,2], the presence of mycotoxins in food poses a potential threat to health and, in more extreme cases, life of both humans and animals.

To date approximately 400 fungal metabolites have been classified as mycotoxins. Zearalenone (ZEN) and T-2 toxin produced by the *Fusarium* spp. family, are of a particular clinical and economic significance due to their prevalence in food and the variety of toxic actions they may exert [2,3].

Zearalenone, also known as F2 mycotoxin, is mostly synthetized by *Fusarium graminearum*, *culmorum*, *crookwellense*, and *roseum*. It is found ubiquitously in food products, such as barley, oat, wheat, maize, peas, bananas, and bread. This mycotoxin acts by binding to estrogen receptors and affecting steroidogenic enzymes. Even at low doses, ZEN causes an estrogenic effect, resulting in changes in sex hormone cycles, hyperestrogenism, and sterility. Moreover, it can lead to developmental disorders, stunted growth, renal failure, and blood clots [4,5]. ZEN also harms the digestive system by causing cell proliferation and inflammation of the intestinal mucosal layer [5,6]. Previous studies have also shown the negative effects of ZEN on the nervous system, which are related to the presence of estrogen receptors in the brain and the fact that phytoestrogens are able to cross the blood-brain barrier [7,8].

T-2 toxin, which belongs to the trichothecenes family of toxins, is produced by *Fusarium sporotrichioides*, *langsethiae*, *acuminatum*, and *poae* [9]. Similarly to ZEN, it is prevalent in food products, especially in grains such as barley, oats, wheat, maize, rye, sorghum, and rice [10]. The effects exerted by this mycotoxin are mostly cytotoxic and immunosuppressive in nature [11]. T-2 toxin is responsible for a variety of conditions including alimentary toxic aleukia (ATA), inflammatory bowel disease, and disorders of the thymus and spleen [2]. It crosses the blood-brain barrier and, thus, it can also elicit changes in the central nervous system [12].

Given their properties, both ZEN and T-2 toxin may affect the gastrointestinal (GI) tract and the nervous system. On the other hand, it is well known that the enteric nervous system (ENS), located in the wall of the digestive tract, regulates all functions of the stomach and intestines and, together with the intestinal immune system, constitutes the first barrier against toxins found in ingested food. The structure of the ENS depends on animal species. In the porcine intestine the ENS is composed of three intramural ganglionated plexuses: the myenteric plexus (MP), located between the longitudinal and circular muscle layers; the outer submucous plexus (OSP), near the circular muscle layer; and, lastly, the inner submucous plexus (ISP), positioned between the muscularis mucosa and lamina propria [5,13].

The enteric plexuses consist of millions of neurons, which play different roles and express a range of neurotransmitters and/or neuromodulators [14]. One of the substances expressed by enteric nerve cells is calcitonin gene related peptide (CGRP), which to date has been found in the ENS and extrinsic innervation of the GI tract of several mammal species, including humans [15,16,17,18]. Within the intestine CGRP is regarded as the key neurotransmitter and/or neuromodulator of the ENS participating in sensory and nociceptive transmission and a marker of the intrinsic primary afferent neurons [19,20]. It is also known that this substance inhibits gastric acid secretion, increases mesenteric blood flow, protects intestinal mucosa and, possibly, exerts relaxatory effects on the gastrointestinal muscle layer [21,22,23]. Moreover, some studies show that CGRP is involved in the regeneration of nervous tissue after injury [24].

Despite the considerable amount of information available on the impact of ZEN and T-2 toxin on living organisms [9], many aspects of their activity remain unknown. One of them is the influence of low doses of these toxins on the ENS. Admittedly, whilst the neurotoxic activity of ZEN and T-2 toxin is not considered to be the most critical effect of these substances, previous studies described the negative impact of them on neuronal cells [7,25]. Moreover both ZEN and T-2 may act on the intestine by different mechanisms which, first of all, include estrogenic activity (in the case of ZEN) and immunological and/or hematological effects (in the case of T-2 toxin) [9,10,26]. These mechanisms of action may in turn impact the ENS [27]. The knowledge connected with the influence of low doses of mycotoxins on enteric neurons is fragmentary and incomplete [5,6,13], although it is known that the ENS is able to undergo structural, functional or chemical changes as a result of adaptive or reparative processes in response to various pathological and toxicological agents [13,28]. The impact of ZEN and T-2 toxin on CGRP distribution in the ENS has not been studied at all, even though this peptide seems to be an important neuronal factor involved in regulatory processes linked to estrogenic and/or immunological activity [29,30].

Therefore, the aim of the present study was to investigate the changes in CGRP-positive nervous structures of the ENS in the porcine colon after the administration of low doses of ZEN and T-2 toxin. Due to the significant sensitivity of the ENS to the presence of harmful agents in the GI tract [28] and the variety of functions that CGRP exerts on the intestinal regulatory processes [20,21,22], these changes may represent the first subclinical symptoms of the damage caused by the mycotoxins studied. The obtained results will enable the development of knowledge about the actions of low doses of ZEN and T-2 toxin on living organisms this study is of particular interest, given that the pig is considered to be an excellent animal model of human physiology [31].

## 2. Results

### 2.1. CGRP-Like Immunoreactivity

During the present investigation, CGRP-LI nervous structures in the porcine descending colon were observed under physiological conditions, as well as after the administration of zearalenone and T-2 toxin (Table 1).

**Figure 1:**
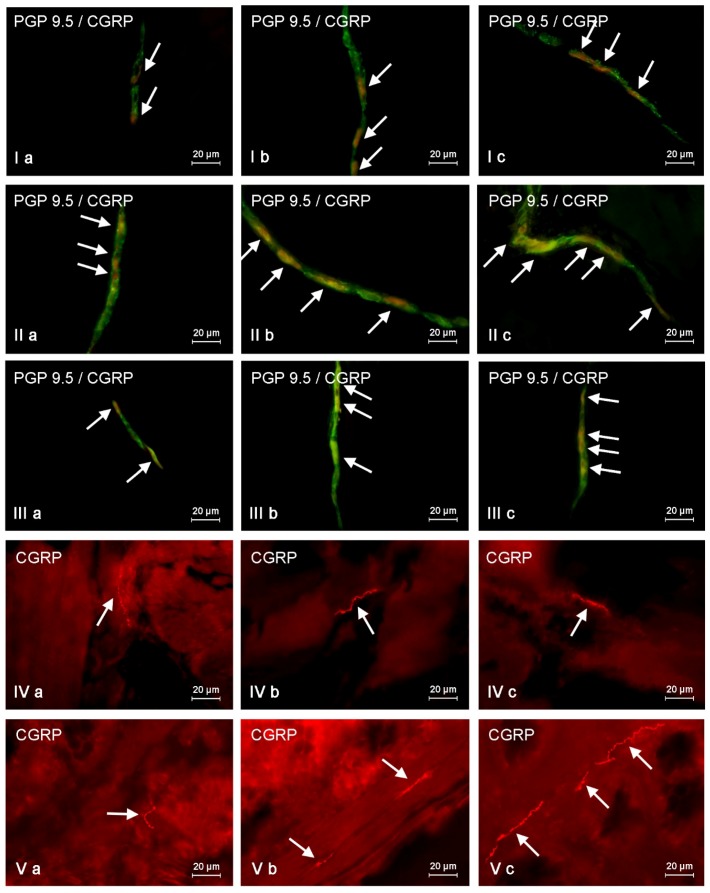
Distribution pattern of nervous structures immunoreactive to protein gene-product 9.5 (PGP 9.5)—used as a panneronal marker and calcitonin gene-related peptide (CGRP) in the wall of porcine descending colon under physiological conditions (**a**), and after T2-toxin (**b**) and zearalenone (**c**) administration; **I**—myenteric plexus; **II**—outer submucous plexus; **III**—inner submucous plexus; **IV**—circular muscle layer and **V**—submucous/mucous layer. CGRP-positive neurons (**I**,**II**,**III**) and nerve fibers (**IV**,**V**) are indicated by arrows. Images **I**, **II**, and **III** are composites of merged images taken separately from green (PGP 9.5) and red (CGRP) fluorescent channels. Images **IV** and **V** are performed using a single immunofluorescence technique.

In physiological conditions (control group) a relatively high percentage of CGRP-LI neurons was observed in all types of enteric plexuses (Figure 1). The greatest number of these neurons was noted in the ISP, where CGRP-positive perikarya amounted to 40.06% ± 1.94% of all cells immunoreactive to protein gene product 9.5 (PGP 9.5), which was used as a panneronal marker. In the MP and OSP these values came out 26.79% ± 1.26% and 38.57% ± 4.12%, respectively.

Overall, both mycotoxins studied caused an increase in the percentage of CGRP-LI neurons present in all types of enteric plexuses (Table 1). However, the changes were more pronounced after ZEN administration (Figure 1). The highest increase in the percentage of CGRP-positive neurons, relative to control animals, was observed in the MP and the ISP of ZEN group (Table 1), where these values amounted to 45.95% ± 3.41% and 59.39% ± 1.64%, respectively. (The increase of about 20 percentage points, pp). The only plexus in which the proportion of the CGRP-positive enteric neurons was not increased in the presence of a mycotoxin was the OSP in animals dosed with T-2 toxin. The changes observed in this condition were not statistically significant (compared to the values observed in control pigs).

Contrary to neuronal cells, changes in the density of colonic CGRP-positive nerve fibers caused by both mycotoxins studied were less pronounced. These fibers were relatively thin and rare (Figure 1) both in the control and experimental groups. The increase in the density of mucosal nerves immunoreactive to CGRP was noted after ZEN and T-2 administration, nerve fibers were located in the muscular layer, where observed changes were not statistically significant (Table 1). In the case of intraganglionic CGRP-LI nerve fibers, the mycotoxin-induced fluctuations were also sparsely visible (Table 1) and limited to the MP (in T-2 and ZEN groups) and the OSP (in the ZEN group only).

### 2.2. Co-Localization of CGRP with Other Neuronal Active Substances

**Figure 2:**
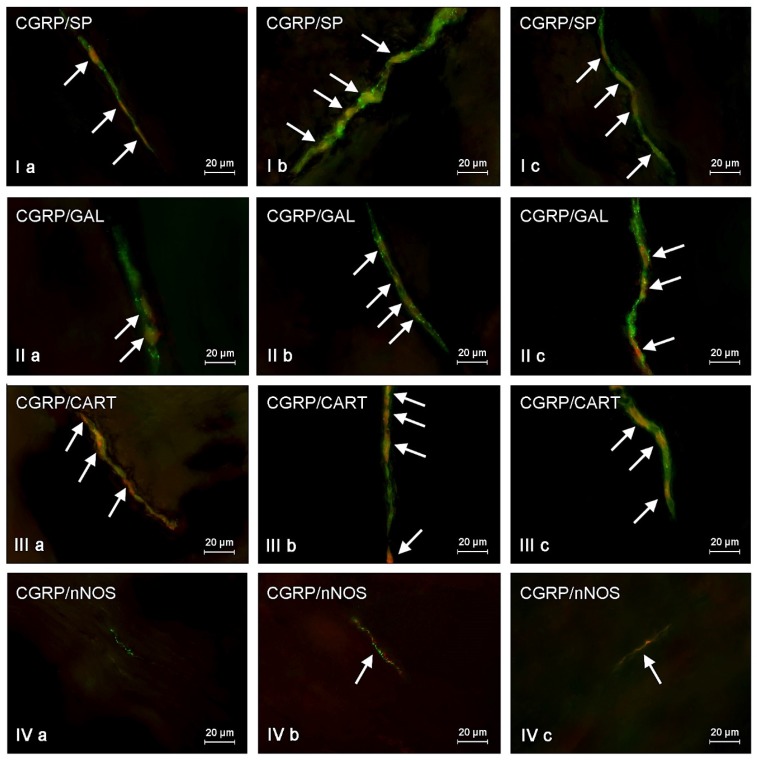
Representative images of co-localization of calcitonin gene-related peptide (CGRP) with other neuronal active substances in the neurons of myenteric plexus (**I**,**II**,**III**) and nerve fibers scattered in the circular muscle layer (**IV**) of the porcine descending colon under physiological conditions (**a**), and after T2-toxin (**b**) and zearalenone (**c**) administration. **I**—co-localization of CGRP with substance P (SP); **II**—co-localization of CGRP with galanin (GAL); **III**—co-localization of CGRP with cocaine- and amphetamine-regulated transcript (CART) peptide; **IV**—co-localization of CGRP with neuronal isoform of nitric oxide synthase (nNOS). Images are composites of merged images taken separately from green (CGRP) and red (other substances studied) fluorescent channels. Nervous structures, where CGRP co-localizes with other substances, are indicated by arrows.

**Figure 3:**
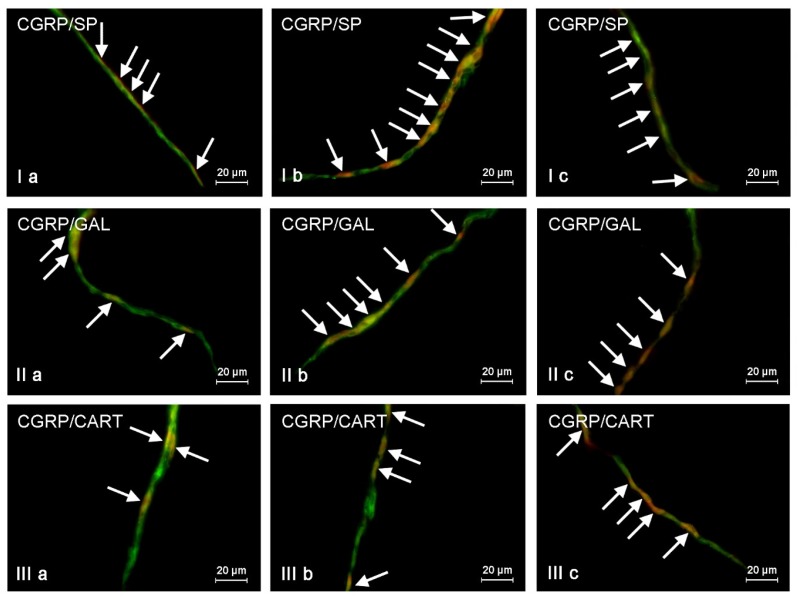
Representative images of co-localization of calcitonin gene-related peptide (CGRP) with other neuronal active substances in the neurons of the outer submucous plexus in the wall of porcine descending colon under physiological conditions (**a**), and after T2-toxin (**b**) and zearalenone (**c**) administration. **I**—co-localization of CGRP with substance P (SP); **II**—co-localization of CGRP with galanin (GAL); **III**—co-localization of CGRP with cocaine- and amphetamine- regulated transcript (CART) peptide. Images are composites of merged images taken separately from green (CGRP) and red (other substances studied) fluorescent channels. Neurons, where CGRP co-localizes with other substances are indicated by arrows.

**Figure 4:**
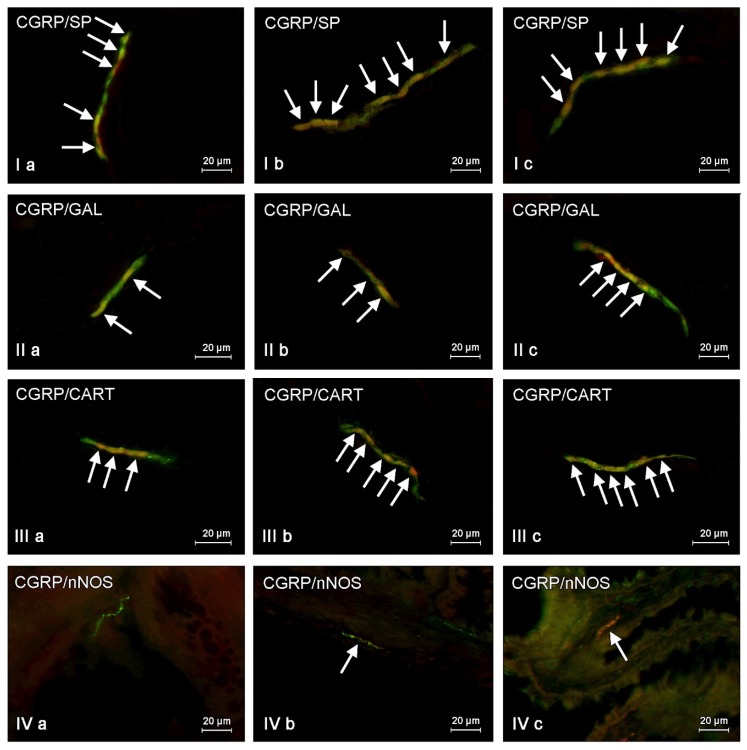
Representative images of co-localization of calcitonin gene related peptide (CGRP) with other neuronal active substances in the neurons of inner submucous plexus (**I**,**II**,**III**) and nerve fibers scattered in submucous/mucous layer (**IV**) of the porcine descending colon under physiological conditions (**a**), and after T2-toxin (**b**) and zearalenone (**c**) administration. **I**—co-localization of CGRP with substance P (SP); **II**—co-localization of CGRP with galanin (GAL); **III**—co-localization of CGRP with cocaine- and amphetamine- regulated transcript (CART) peptide; **IV**—co-localization of CGRP with neuronal isoform of nitric oxide synthase (nNOS). Images are composites of merged images taken separately from green (CGRP) and red (other substances studied) fluorescent channels. Nervous structures, where CGRP co-localizes with other substances are indicated by arrows.

In the present study the co-localization of CGRP with other active substances, such as substance P (SP), neuronal isoform of nitric oxide synthase (nNOS, used as a marker of nitrergic neurons), galanin (GAL), vesicular acetylcholine transporter (VAChT, used as a marker of cholinergic neurons), and cocaine- and amphetamine- regulated transcript peptide (CART) was observed both in the control animals and following the administration of the two mycotoxins (Figure 2, Figure 3 and Figure 4). The degree of co-localization of CGRP with particular substances depended on the type of enteric plexus and class of neuronal factor studied.

The majority of CGRP-positive enteric neurons were also immunoreactive to substance P (Table 2). SP was present in 56.36% ± 0.73%, 64.9% ± 3.9%, and 64.91% ± 4.93% of all CGRP-positive neurons in the MP (Figure 2), the OSP (Figure 3), and the ISP (Figure 4), respectively. Contrary to neuronal cell bodies, the degree of co-localization of CGRP and SP in the nerve fibers in the muscular and mucosal layers was much lower. Only 27.66% ± 1.43% of all the intramuscular nerves immunoreactive to CGRP were also SP-positive. In the mucosal layer this value amounted to 14.79% ± 0.87%.

ZEN and T-2 toxin caused a significant increase in the degree of co-localization of CGRP and SP in neuronal cell bodies and nerve fibers. The extent of the mycotoxic effect was comparable in the two groups (Table 2, Figure 2, Figure 3 and Figure 4). The highest percentage of CGRP-positive neurons, which also contained substance P, as well as the highest increase in the absolute number of such cells, was observed in the ISP of animals after ZEN administration (Figure 4). This value in ZEN group amounted to 91.3% ± 3.46% (increase of about 25 pp compared with control animals). On the other hand the differences in the degree of co-localization of CGRP and SP in the OSP neurons (Figure 3) between C and ZEN groups were not statistically significant (Table 2).

Another substance that was observed in CGRP-LI neurons was nNOS (Table 3). The degree of co-localization of these two factors under physiological conditions was slightly lower than that observed for CGRP and SP, and varied between 38.65% ± 1.43% in the MP and 58.94% ± 1.11% in the ISP. In contrast to the other active neuronal factors studied, CGRP and nNOS did not co-localize in the intramuscular (Figure 2IVa) and intramucosal (Figure 4IVa) nerve fibers of the control animals. Interestingly, in the pigs after administration of T-2 toxin and zearalenone a small number of CGRP+/nNOS+ nerves appeared both in the circular muscle (Figure 2IVb,IVc) and in the submucous/mucous layers (Figure 4IVb,IVc). The most notable changes in the degree of co-localization of CGRP and nNOS in the enteric neurons were observed in the MP and the OSP in pigs after ZEN administration, where nNOS was found in 51.54% ± 3.71% and 60.69% ± 2.45% of all CGRP-LI cells, respectively. The increase in both mentioned above plexuses achieved about 13 pp compared with values observed in control animals. The differences in the percentage of CGRP+/nNOS+ neurons in the ISP between the control animals and pigs after administration of both mycotoxins were less visible (Table 3).

The degree of co-localization of CGRP and GAL was similar to that observed in case of CGRP and nNOS (Table 4). In the control animals, the percentage of CGRP+/GAL+ neuronal cells ranged from 37.42% ± 1.07% of all CGRP-LI neurons in the MP (Figure 2) to 52.82% ± 5.39% in the ISP (Figure 4). Overall, both toxins caused an increase in the expression of GAL in neurons immunopositive to CGRP. The most notable changes were observed in the MP (Figure 2) and the OSP (Figure 3) of animals after T-2 toxin administration, and amounted to 56.65% ± 1.95% and 62.05% ± 2.92%, respectively. In these both plexuses the increase of CGRP-positive cells, which were also immunoreactive to GAL, had a value about 20 pp in relation to control animals. In contrast, the changes caused by both mycotoxins in the degree of co-localization of CGRP and GAL in the ISP neurons were not statistically significant (Figure 4). Administration of ZEN and T-2 toxin contributed to an increase in the expression of GAL in CGRP-positive intramuscular and intramucosal nerves (Table 4).

The co-localization of CGRP and CART (Table 5) was also noted during the present investigation. Under physiological conditions the presence of CART was observed in a significant number of CGRP-LI neuronal cell bodies within all types of enteric plexuses. The degree of co-localization of these two substances was particularly high in the ISP (Figure 4), where it reached 62.18% ± 1.12%. Moreover, both mycotoxins studied caused a statistically significant increase in the expression of CART in CGRP-positive neurons within all types of enteric plexuses (Figure 2, Figure 3 and Figure 4). The most visible changes (compared to the control animals) were observed in the OSP (increase of about 15 pp) and the ISP (increase of about 10 pp) in the ZEN administered animals (Table 5).

The degree of co-localization of CGRP and CART in the nerve fibers was also high. In the control animals it amounted to 63.01% ± 1.96% and 54.8% ± 5.61% in the submucous/mucous and circular muscle layers, respectively. Furthermore, ZEN administration caused a statistically significant increase in the expression of CART in CGRP-positive intramucosal and intramuscular nerve fibers (Table 5).

A large number of CGRP-LI enteric neuronal cells observed during the present study were cholinergic neurons (Table 6). In the control animals the percentage of CGRP+/VAChT+ neurons ranged from 53.84% ± 0.57% of all CGRP-positive cells in the MP to 63.63% ± 1.29% in the ISP. Intramuscular and intramucosal nerve fibers also showed a high degree of co-localization of CGRP and VAChT (Table 6). However, contrary to the co-localization of CGRP with the other neuronal active substances studied, mycotoxins generally did not change the expression of VAChT in the CGRP-positive enteric nervous structures.

## 3. Discussion

This study demonstrates that CGRP is present in neurons and nerves located in the porcine descending colon both under physiological conditions and after mycotoxin administration, and the number of CGRP-LI neurons clearly depends on the type of enteric plexuses.

These observations are generally in agreement with previous studies, where CGRP-positive structures of the ENS have been described mainly in the stomach and small intestine in a wide range of mammal species, including humans [16,32,33,34]. Moreover, significant interspecies differences in the distribution of enteric neurons immunoreactive to CGRP have been established [32,33,34], which can suggest that exact roles of this peptide are different in various animal species. In contrast, the knowledge concerning the distribution of CGRP in the colonic ENS is relatively scarce and essentially limited to humans and rats [35,36]. It should be pointed out that some similarities between humans [16,33,37] and pigs (this study) have been observed. This fact may confirm previously-described anatomical and physiological resemblances of the ENS between these species, which cause pigs to currently be considered as an optimal laboratory animal for studies on the human GI tract [31].

A relatively high number of CGRP-positive enteric neurons observed during the present study can suggest important and multidirectional functions of this substance in intestinal regulatory processes, what is confirmed by previous investigations. First of all, this peptide takes part in the conduction of sensory and pain impulses [19,20], but it also increases a blood flow in mesenteric vessels [21], inhibits gastric acid secretion, and regulates the absorption of nutrients from the gut [22]. Other important functions of CGRP are the ability to stimulate the secretion of other neurotransmitters, including somatostatin and nitric oxide [38], and the protection of endothelial cells against damage [24]. Moreover, although this peptide is not classified as a typical peristaltic regulatory factor, it has a relaxatory effect on the intestinal muscles [23]. In spite of the above-mentioned functions of CGRP in the GI tract, a large number of aspects connected with the distribution and roles of these peptide in the ENS still remain unknown. One of them is the reaction of CGRP-positive enteric neurons on toxins in food.

During the present investigation both ZEN and T-2 toxin generally caused the increase in the number of CGRP-LI enteric nervous structures. This fact suggests that this peptide plays many important roles in the ENS, not only in physiological conditions, but also under intoxication. The observed changes may result from various reasons. One of them is the direct action of mycotoxins studied on the neuronal cells, connected with the damage of mitochondria, in the case of T-2 toxin, or the action on estrogen receptors and genotoxic effects (including the DNA fragmentation, apoptosis and chromosome aberrations), in the case of ZEN [4,9,10]. Nonetheless, it should be pointed out that, in spite of the relatively well-described influence of ZEN and T-2 toxin on various internal organs, their neurotoxic effects, especially in low doses, are still disputable and often considered of no significance [9,10]. On the other hand, some previous studies described clear effects of these substances on neurons in the central nervous system. Namely, it is known that ZEN may exhibit the negative influence on brain neurons, what is probably connected with the decrease of brain calcium-binding proteins levels [7] and/or oxidative stress mechanisms [8]. Additionally, this toxin may have adverse impacts on glial cells [39].

In turn, T-2 toxin has been described as a factor that may cause the oxidative damage in the mouse brain [25] and change the expression of monoamines in various regions of the rat brain [12]. Moreover, it is known that T-2 toxin can induce the apoptosis of neurons in fetal and adult brains [40], and the administration of this toxin in rats results in the inhibitory effects on motor activity [41]. The mentioned changes are probably connected with the influence of T-2 toxin on the permeability and impairment of the blood-brain barrier [12].

Until now, the neurotoxic activities of ZEN and T-2 toxin on the enteric nervous system have not been studied, but it can be assumed that effects of these toxins on enteric neurons are similar to those observed in the central nervous system. Admittedly, the neurotoxicity of low doses of mycotoxins studied has not been confirmed so far, but the ENS may be more sensitive to activity of these substances due to the direct exposure to toxins in food. Thus, the changes observed during the present study may result from the neurotoxic activity of ZEN and T-2 toxin and can be an effect of neuroprotective and/or neuro-adaptive actions of CGRP. Two facts confirm this supposition. Firstly, it is well known that the expression of neuroprotective factors in the ENS increases during pathological processes [5,6,42,43], and the growth of CGRP-like immunoreactivity has been observed during both the present study and in previous investigations on other fragments of the digestive tract [44]. Secondly, CGRP affects the release of nitric oxide, whose neuroprotective activity is relatively well surveyed [45,46,47]. The cooperation of CGRP and nitric oxide has been also confirmed during the present study, where the relatively high degree of the co-localization of CGRP with nNOS (a marker of nitrergic neurons) has been observed in all types of enteric plexuses, especially after the mycotoxins administration. Moreover, it is relatively well established that CGRP expression may change during the neurotoxicity in various parts of the nervous system and/or neuropathies of different origins [48,49,50,51,52], which can confirm neuroprotective and adaptive functions of this neuronal factor.

Of course, the changes noted in the present study may arise not only from direct neurotoxic effects of mycotoxins studied on the ENS, but they can also be a result of other processes. In the case of zearalenone, the reaction of enteric neurons may be connected with the commonly known ability of this toxin to bind to estrogen receptors, which are widely distributed in various internal organs, among others, within the whole gastrointestinal tract [53,54,55]. Previous studies reported that estrogen and other substances binding to estrogen receptors (and, therefore, also ZEN) may regulate various gastrointestinal functions that depend on the fragment of the digestive tract and include the intestinal motility, ion transport, and intestinal endocrine system activity [54]. On the other hand, it is well established that the above-mentioned functions of the GI tract are regulated by the ENS. Therefore, the changes observed during the present study may result from the estrogenic activity of ZEN. It is the more likely that CGRP is the factor participating in regulatory processes of the intestinal motility and excretive activity [21]. Moreover, it is known that nitric oxide is involved in the modulating of the colonic motility by estrogen receptors [56] and, during the present study, the changes in the co-localization of CGRP and nNOS were observed.

The other reason of the changes noted in animals after ZEN administration may be the influence of this substance (even in low doses) on the gut immune system. Namely, previous studies described that ZEN causes a significant increase in levels of IL-1, IL-2, IL-10, and IFNγ produced by immune cells located in the intestine [57]. Moreover, it is known that low doses of ZEN may change the sensitivity of intestinal T and B lymphocytes to lipopolysaccharides [58]. Due to the fact that the intestinal immunological system closely cooperates with the ENS [27], the above-mentioned actions of ZEN may cause fluctuations in neurochemical characterization within enteric neurons observed during the present investigation.

Mechanisms connected with immunological processes may also be at the heart of changes observed in animals after T-2 toxin administration. The main, and relatively well-known, effect of T-2 toxin is the inhibition of protein synthesis [10] which, among others, may manifest itself by the influence of this toxin on the immunological system [10,59]. Previous studies described that this substance reduces the proliferative response of lymphocytes and impairs the antibody production [9,26]. Moreover, T-2 toxin may hinder the maturation of dendritic cells [60] and suppresses the immune response to bacterial infections [9]. T-2 toxin-induced modulatory effects on the immunological system have been also described within the GI tract, where low subclinical doses of this substance caused the changes in immune cells polarization, T cell memory, and humoral immunological response mediated by B lymphocytes in the ileal Peyear’s patches [59]. In turn, the influence of T-2 toxin on the immunological system may be connected with hematological effects—the second-most important toxic activity of this substance—which includes the decrease in the number of red blood cells, leukopenia, and changes in the composition of blood serum [9,10].

On the grounds of close cooperation between the immunological system and enteric neurons in the maintenance of intestinal homeostasis [27], mentioned above effects of T-2 toxin on immune cells would be the cause of fluctuation in CGRP-like immunoreactivity observed during the present study. It is the more likely that CGRP is known as an important neuronal factor involved in modulation of immune responses [29]. It is also possible that T-2 toxin-induced changes in the blood composition may have an impact on intestinal blood flow which, in turn, is regulated by CGRP [21].

Nonetheless, fluctuations in CGRP-like immunoreactivity noted in the present study may also result from the indirect activity of both toxins studied, connected with inflammatory processes [61,62]. It is possible that the observed changes, first of all, are associated with relatively well-known participation of CGRP in sensory and pain conduction [19,20]. On the other hand, low doses of the mycotoxins studied during the present experiment are not expected to be a reason for significant pain. Thus, fluctuations in CGRP-like immunoreactivity may be connected with the regulation of blood flow of intestinal vessels and/or the mucosal layer protection of during subclinical inflammation [21,24].

The presented data above shows that changes noted in the present study may result from various mechanisms. The detailed explanation of them is the more difficult due to the fact that CGRP is present in different types of enteric neurons and may co-localize with various other neurochemical factors. On the other hand, it is known that substances co-localizing in the same neuronal cell usually play similar functions, and results of the present study can be important for the explanation of detailed roles of CGRP in the ENS, not only under physiological conditions, but also during mycotoxin intoxication. It should be pointed out that the present study is the first exact characterization of the neurochemical coding of CGRP-positive enteric neurons in the porcine digestive tract. Hence, the short description of neuronal factors, which co-localized with CGRP, seem to be fully justified.

First of all, CGRP-positive enteric neurons observed during the present investigation also contained VAChT, used as a marker of acetylcholine, which is the main excitatory neuromediator within the ENS [63]. The second substance noted in CGRP-LI enteric neurons was SP, which (like CGRP) is associated with transmission of the sensory and pain information [64]. It is also involved in the regulation of the intestinal motility and secretion, as well as neuroprotective effects [64,65]. The next substance, which was co-localized with CGRP was nNOS—the marker of nitrergic neuronal cells. In addition to relatively good established neuroprotective functions of nitric oxide [66], this gaseous transmitter is known as a very important inhibitory factor, which affects the intestinal muscles, as well as secretion of electrolytes and hormones within the GI tract [45,46]. Contrary to the above-mentioned factors, the gastrointestinal functions of GAL and CART, which were also observed in CGRP-positive enteric neurons, are more obscure. It is known that GAL, depending on the fragment of the GI tract and animal species studied, can play various, often contradictory, roles [42]. In turn, CART may affect the intestinal motility, but the mechanisms of this action, due to undefined receptors of this peptide, are unknown [13,67]. It should be pointed out that ZEN and T-2 toxin caused the increase not only in the percentage of CGRP-positive cells, but also changes in the degree of co-localization of this peptide with majority of mentioned above neuronal factors. It can suggest the interaction between CGRP and other active substances and their neuroprotective functions in the ENS during mycotoxin poisoning.

To sum up, the obtained results show that even low doses of ZEN and T-2 toxin may affect the neurochemical profile of neurons in the ENS of large intestine. On the other hand, CGRP seems to be an important factor in regulatory processes connected with mycotoxins activity. However, a wide range of substances, which co-localized with CGRP, and changes in the neurochemical profile of CGRP-LI enteric neurons suggest that functions of this peptide in the ENS during mycotoxins intoxication are multidirectional and complex. Moreover, fluctuations in CGRP-like immunoreactivity may result from various mechanisms connected with mycotoxins activity and can be caused by changes in the transcriptional, translational, or metabolic levels of CGRP synthesis and/or disturbances in the intraneuronal transport. Due to these ambiguities the total explanation of CGRP functions in the ENS during intoxication requires further investigations.

## 4. Materials and Methods

The present investigation was performed on 15 immature female pigs (eight weeks of age, 18–20 kg body weight) of the large White Polish breed. Animals were randomly divided into three groups (five pigs in each group): control group (C Group), where empty gelatin capsules were administered, and two experimental groups, where capsules containing T-2 toxin (T-2 Group) or zearalenone (ZEN Group) were administered. T-2 toxin was given with a dose of 12 µg/kg body weight (b.w.) per day, and ZEN, with a dose of 6 µg/kg b.w. per day. According to European Food Safety Authority 2014 [68] a dose of T-2 toxin was clearly lower than the lowest-observed-adverse-effect level (LOAEL) which, for pigs, is set at 29 µg/kg b.w. per day, and a dose of ZEN was significant lower than the no-observable-adverse-effect-level (NOAEL) for estrogenic effects, which amounts to 10 µg/kg b.w. per day. Capsules in all groups of animals were administered per os, once daily before the morning feeding, for 42 days. Pigs were fed using the commercial all-mash feed for piglets of known composition, called “WIGOR 3” (WIPASZ S.A, Olsztyn, Poland). Moreover, in order to exclude accidental mycotoxin contamination, feed was tested for the presence of the following substances: Aflatoxin B1, T-2 toxin, ochratoxin A (OTA), ZEN, alpha-zearalenol (α-ZEL), and deoxynivalenol (DON). These tests were performed with common separation techniques using the immunoaffinity columns (Afla-TestR P Aflatoxin testing system, G1010, VICAM, Watertown, MA, USA; T-2-TestTM HPLC Mycotoxin Testing System G1028, VICAM, Watertown, MA, USA; Ochra-TestTM WB Mycotoxin Testing System, G1033, VICAM, Watertown, MA, USA; Zearala-Test™ Zearalenone Testing System, G1012, VICAM, Watertown, MA, USA; DON-Test™ DON Testing System, VICAM, Watertown, MA, USA) and high-performance liquid chromatography (HPLC) (Agilent Technologies, Santa Clara, California, USA, type 1050 and 1100) with fluorescent and/or UV detection techniques. None of the above-mentioned substances were present in tested feed.

During the experiment the pigs were kept under standard laboratory conditions, and all experimental procedures were performed according to the instructions of the Local Ethical Committee for Animal Experimentation in Olsztyn (Poland) (decision from 28 November 2012, identification code 73/2012/DTN).

On the experimental day 43 all pigs were pre-medicated with Stressnil (Janssen, Belgium, 75 μL/kg of body weight given intravenously) and after 15 min euthanized using an overdose of sodium thiopental (Thiopental, Sandoz, Kundl-Rakúsko, Austria). Immediately after euthanasia approximately 2-cm-long fragments of descending colon (the same fragments from all animals) were collected and fixed in a solution of 4% buffered paraformaldehyde (pH 7.4) for one hour. Later on, the tissues were rinsed in phosphate buffer (0.1 M, pH 7.4, at 4 °C) for three days (with daily exchange of buffer), inserted into 18% phosphate-buffered sucrose and storage at 4 °C for at least two weeks. Then, the fragments of descending colon were frozen at −22 °C, cut perpendicularly to the lumen of the GI tract into 14-µm-thick sections using microtome (Microm, HM 525, Walldorf, Germany), and fixed on glass slides.

The slices were subjected to standard double-labelling immunofluorescence technique, which has been described previously by Gonkowski et al. [69].

In short, this method was performed as follows: Frozen sections of the descending colon on glass slides were dried for 45 min. at room temperature (rt) and incubated with blocking solution containing 10% goat serum, 0.1% bovine serum albumin (BSA), 0.01% NaN_3_, Triton X-100, and thimerosal in PBS (1 h, rt). Then, samples were incubated (overnight; rt, in a humid chamber) with a mixture of two antibodies raised in different species and directed towards CGRP and one of the other substances studied, i.e., PGP 9.5 (used here as pan-neuronal marker), SP, GAL, nNOS (used as a marker of nitrergic neurons), VAChT (used here as marker of cholinergic neurons) and CART (the precise specification of primary antisera is presented in Table 7). Complexes of primary antibodies and appropriate antigens were visualized by incubation (1 h, rt) with species-specific secondary antisera (Table 7) conjugated to Alexa fluor (1 h, rt). Each step of the immunofluorescence technique was followed by rinsing the sections with PBS (3 × 10 min, pH 7.4).

Specificity of the labelling was verified by standard control procedures, including pre-absorption of primary antisera with appropriate antigens, as well as omission and replacement tests. These procedures completely eliminated specific stainings.

Tissues were viewed using an Olympus BX51 microscope equipped with epi-fluorescence and appropriate filter sets. Only neurons with clearly-visible nuclei were included in the present experiment. To evaluate the percentage of CGRP-LI neurons in relation to all enteric neuronal cells, at least 500 PGP-9.5-labeled cell bodies in particular types of enteric plexuses (MP, OSP, and ISP) in each animal were examined, and the number of neurons immunoreactive to PGP 9.5 was treated as 100%. In the case of investigation on the co-localization of CGRP with other substances, at least 150 CGRP-positive cell bodies in particular types of enteric ganglia were examined for immunoreactivity to particular neuronal factors. In these studies, CGRP-positive neurons were considered as representing 100%. The obtained data were pooled and presented as the mean ± SEM. To prevent double counting of the same neurons, the evaluated sections of the descending colon were located at least 150 µm apart.

Moreover, an arbitrary semi-quantitative method was used to determine the density of intraganglionic CGRP-positive nerve fibers. This method was based on the scale from (−), indicating the absence of CGRP-LI nerves, to (++++), depicting a very dense meshwork of fibers studied. The density of CGRP-LI nerves were evaluated in all enteric ganglia, where the percentage of CGRP-positive neurons were studied (at least 60 ganglia of each type from each animal). Then the total number of (+) from all evaluated ganglia was summed and divided by the number of ganglia. The obtained numbers rounded to the integers showed the median quantity of (+) in the particular types of ganglia.

In turn, the evaluation of the density of CGRP-LI nerves in the muscular and mucosal layers was based on the counting of them per microscopic observation field (0.1 mm^2^). The number of nerve fibers were evaluated in four fragments of the descending colon per animal (in five fields per section) and the obtained data were pooled and presented as the mean ± SEM.

Another method is used to denote the neurochemical characterization of CGRP-positive nerves in the muscular and mucosal layers. Namely, at least 100 nerves immunoreactive to CGRP were evaluated for immunoreactivity to each of other neuronal factors studied, and the obtained data were also pooled and presented as the mean ± SEM. To prevent double counting of nerve fibers, in all above-mentioned methods, the evaluated sections of the colon were located at least 250 µm apart. All pictures were captured by a digital camera connected to a PC. Statistical analysis was made with the one-way ANOVA test (Statistica 9.1, StatSoft, Inc., Cracow, Poland) and differences were considered statistically significant at *p* ≤ 0.05.

## Figures and Tables

**Table 1:** CGRP-LI perikarya and nerve fibers in the porcine descending colon.

Fragment of Colonic Wall		Control Group	T-2 Group	ZEN Group
Circular muscle layer (1)		0.44 ± 0.02	0.73 ± 0.14	0.51 ± 0.03
Myenteric plexus	Cell bodies (2)	26.79 ± 1.26 *	42.57 ± 3.01 *	45.95 ± 3.41 *
	Nerve fibres (3)	+	++	+++
Outer submucous plexus	Cell bodies (2)	38.57 ± 4.12 *	50.09 ± 2.57	53.98 ± 2.18 *
	Nerve fibres (3)	+	+	++
Inner submucous plexus	Cell bodies (2)	40.06 ± 1.94 *	52.45 ± 3.73 *	59.39 ± 1.64 *
	Nerve fibres (3)	+	+	+
Mucosal layer (1)		1.38 ± 0.34 *	2.66 ± 0.22 *	3.58 ± 0.7 *

(1) Average number of CGRP-LI nerve fibers per area studied (mean ± SEM); (2) the percentage of CGRP-LI neurons (mean ± SEM) in relation to neurons stained for PGP 9.5 (used as pan neuronal marker); (3) the density of intraganglionic CGRP-LI nerve fibers presented in arbitrary units on the scale from (−), indicating the absence of CGRP-LI nerves, to (++++), depicting a very dense meshwork of fibers studied. Statistically significant differences (*p* ≤ 0.05) between control group and T2 group, as well as between the control group and the ZEN group, are marked with *.

**Table 2:** Co-localization of calcitonin gene related peptide (CGRP) with substance P (SP) in the enteric nervous structures of the porcine descending colon.

CGRP/SP			
Fragment of Colonic Wall	Control Group	T2 Group	ZEN Group
Circular muscle layer (1)	27.66 ± 1.43 *	35.85 ± 0.7 *	49.25 ± 2.78 *
Myenteric plexus (2)	56.36 ± 0.73 *	76.82 ± 2.61 *	75.41 ± 3.9 *
Outer submucous plexus (2)	64.9 ± 3.9 *	82.9 ± 3.25 *	78.7 ± 4.55
Inner submucous plexus (2)	64.91 ± 4.93 *	85.73 ± 1.89 *	91.3 ± 3.46 *
Mucosal layer (1)	14.79 ± 0.87 *	29.85 ± 2.27 *	35.95 ± 2.21 *

Values are presented as the relative frequency of SP-LI nerves (1) or cell bodies (2), presented as % (mean ± SEM) in relation to all counted fibers (1) or cell bodies (2) stained for CGRP (CGRP-LI fibers (1) or cell bodies (2) were treated as 100%). Statistically significant differences (*p* ≤ 0.05) between control group and T2 group as well as between control group and ZEN group are marked with *.

**Table 3:** Co-localization of CGRP with neuronal isoform of nitric oxide synthase (nNOS) in the enteric nervous structures of the porcine descending colon.

CGRP/nNOS			
Fragment of Colonic Wall	C Group	T2 Group	ZEN Group
Circular muscle layer (1)	-	9.17 ± 0.32 *	9.26 ± 0.39 *
Myenteric plexus (2)	38.65 ± 1.43 *	43.37 ± 0.82 *	51.54 ± 3.71 *
Outer submucous plexus (2)	48.32 ± 0.67 *	53.17 ± 1.36 *	60.69 ± 2.45 *
Inner submucous plexus (2)	58.94 ± 1.11 *	64.73 ± 1.36 *	64.46 ± 1.93
Mucosal layer (1)	-	5.37 ± 0.92 *	4.81 ± 0.61 *

Values are presented as the relative frequency of nNOS-LI nerves (1) or cell bodies (2), presented as % (mean ± SEM) in relation to all counted fibers (1) or cell bodies (2) stained for CGRP (CGRP-LI fibers (1) or cell bodies (2) were treated as 100%). Statistically significant differences (*p* ≤ 0.05) between control group and T2 group as well as between control group and ZEN group are marked with *.

**Table 4:** Co-localization of CGRP with galanin (GAL) in the enteric nervous structures of the porcine descending colon.

CGRP/GAL			
Fragment of Colonic Wall	C Group	T2 Group	ZEN Group
Circular muscle layer (1)	36.37 ± 1.41 *	47.83 ± 2.17 *	49.98 ± 2.29 *
Myenteric plexus (2)	37.42 ± 1.07 *	56.65 ± 1.95 *	44.1 ± 1.12 *
Outer submucous plexus (2)	44.92 ± 2.03 *	62.05 ± 2.92 *	57.34 ± 2.86 *
Inner submucous plexus (2)	52.82 ± 5.39	64.57 ± 2.32	63.53± 2.27
Mucosal layer (1)	28.07 ± 0.36 *	41.94 ± 1.65 *	46.74 ± 2.27 *

Values are presented as the relative frequency of GAL-LI nerves (1) or cell bodies (2), presented as % (mean ± SEM) in relation to all counted fibers (1) or cell bodies (2)stained for CGRP (CGRP-LI fibers (1) or cell bodies (2) were treated as 100%). Statistically significant differences (*p* ≤ 0.05) between control group and T2 group as well as between control group and ZEN group are marked with *.

**Table 5:** Co-localization of CGRP with cocaine- and amphetamine-regulated transcript peptide (CART) in the enteric nervous structures of the porcine descending colon.

CGRP/CART			
Fragment of Colonic Wall	C Group	T2 Group	ZEN Group
Circular muscle layer (1)	63.01 ± 1.96 *	68.66 ± 1.65	71.29 ± 1.82 *
Myenteric plexus (2)	41.35 ± 0.45 *	48.28 ± 1.12 *	48.08 ± 0.45 *
Outer submucous plexus (2)	45.74 ± 1.93 *	52.57 ± 1.46 *	60.5 ± 2.66 *
Inner submucous plexus (2)	62.18 ± 1.12 *	66.58 ± 1.08 *	72.95 ± 1.13 *
Mucosal layer(1)	54.8 ± 5.61 *	64.27 ± 3.3	72.83 ± 1.99 *

Values are presented as the relative frequency of CART-LI nerves (1) or cell bodies (2), presented as % (mean ± SEM) in relation to all counted fibers (1) or cell bodies (2)stained for CGRP (CGRP-LI fibers (1) or cell bodies (2) were treated as 100%). Statistically significant differences (*p* ≤ 0.05) between control group and T2 group as well as between control group and ZEN group are marked with *.

**Table 6:** Co-localization of CGRP with vesicular acetylcholine transporter (VAChT) in the enteric nervous structures of the porcine descending colon.

CGRP/VAChT			
Fragment of Colonic Wall	C Group	T2 Group	ZEN Group
Circular muscle layer (1)	74.5 ± 0.77	76.25 ± 1.9	74.96 ± 0.95
Myenteric plexus (2)	53.84 ± 0.57	54.01 ± 0.45	54.76 ± 0.68
Outer submucous plexus (2)	54.74 ± 0.83	54.69 ± 0.67	54.71 ± 1.04
Inner submucous plexus (2)	63.63 ± 1.29	62.26 ± 0.49	64.43 ± 1.24
Mucosal layer(1)	77.25 ± 0.38 *	79.06 ± 0.53 *	76.88 ± 0.3

Values are presented as the relative frequency of VAChT-LI nerves (1) or cell bodies (2), presented as % (mean ± SEM) in relation to all counted fibers (1) or cell bodies (2)stained for CGRP (CGRP-LI fibers (1) or cell bodies (2) were treated as 100%). Statistically significant differences (*p* ≤ 0.05) between control group and T2 group as well as between control group and ZEN group are marked with *.

**Table 7:** List of antisera and reagents used in immunohistochemical investigations.

**Primary Antibodies**				
**Antigen**	**Code**	**Species**	**Working Dilution**	**Supplier**
PGP 9.5	7863-2004	Mouse	1:1000	Biogenesis Ltd., Poole, UK
CGRP	T-5027	Guinea Pig	1:1600	Peninsula, San Carlos, CA, USA
CGRP	AB5920	Rabbit	1:1600	Chemicon Int Temecula, OH, USA
SP	8450-0505	Rat	1:1000	Bio-Rad (AbD Serotec), Kidlington, UK
nNOS	AB5380	Rabbit	1:2000	Merck Millipore, Warsaw, Poland
GAL	T-5036	Guinea Pig	1:2000	Peninsula
CART	1-003-61	Rabbit	1:8000	Phoenix Pharmaceuticals, Inc., Belmont, CA, USA
VAChT	H-V006	Rabbit	1:2000	Phoenix Pharmaceuticals
**Secondary Antibodies**				
**Reagents**			**Working Dilution**	**Supplier**
Alexa fluor 488 donkey anti-mouse IgG			1:1000	Invitrogen, Carlsbad, CA, USA
Alexa fluor 488 donkey anti-rabbit IgG			1:1000	Invitrogen
Alexa fluor 488 donkey anti-guinea pig IgG			1:1000	Invitrogen
Alexa fluor 546 donkey anti-mouse IgG			1:1000	Invitrogen
Alexa fluor 546 donkey anti-rabbit IgG			1:1000	Invitrogen
Alexa fluor 546 donkey anti-rat IgG			1:1000	Invitrogen
Alexa fluor 546 donkey anti-guinea pig IgG			1:1000	Invitrogen
